# Memorials to Nurse Cavell

**Published:** 1915-12-04

**Authors:** 


### THE LONDON HOSPITAL.

### Memorials to Nurse Cavell.

At a Court of the London Hospital, held on 1st inst., the report of proceedings supplied the following matters of interest:—

Wounded Soldiers and Sailors.

The Committee has had up to date 3,238 wounded soldiers under its care, with a total number of forty-five deaths. Considering the gravity of many of the cases, this is a remarkably low figure. In the hospital 324 sailors have been treated, and, fortunately, only one of the cases has died. Cases are being admitted from the Dardanelles as well as from France. In view of the advent of winter, entertainments for the convalescent soldiers and sailors have been arranged. These take place twice a week in the library belonging to the Medical College, and are under the management of Mr. George Clark, who kindly offered his services to the Chairman. Many eminent artists have already promised to perform, amongst them Sir John Hare, Mr. H. B. Irving, and Mr. Denis Eadie. Mr. Gerald du Maurier and Mr. Henry Ainley came last week and brought a number of well-known ladies and gentlemen, viz. Mr. Jack Norworth, Miss Margaret Cooper and Miss Gladys Cooper, etc., to assist in giving a most admirable entertainment which was much enjoyed by the wounded soldiers and sailors. Mr. Clark has provided a permanent cinematograph apparatus which has been fixed in the gallery of the library, and he sends an expert every Friday afternoon to give a show. The Committee gratefully acknowledges the great generosity of the Committee of the Young Men's Christian Association, who have lent a recreation hut for the use of the wounded, and which is being built on the lawn.

Some Interesting Items.

The hospital and all hospital property have been insured against risk of damage by aircraft at a cost of £580 under the Government Insurance Scheme. The hospital has lately taken over the management of the dental department, which was formerly part of the medical college, and managed by the college board.

The Grocers' Company's new wards built on the top of the isolation block will be called the "St. Anthony Wards."

The committee is deeply sensible of the generosity of Mrs. Croft, who has lately tiled Queen Victoria children's surgical ward, she having already defrayed the cost of tiling "Buxton" and "David Hughes," with the children's wards. A division of Yarrow Ward has been named the "Helen Croft Ward," as a slight recognition of all the hospital owes to Mrs. Croft's kindness.

The secretary, Mr. E. W. Morris, has returned from the War Office, where he has spent three months in assisting the authorities in procuring drugs, dressings, and surgical instruments.

Mrs. Brinton has presented the hospital with two "Simpson" lamps, which are being installed in the light department.

Mr. Hugh Lett, one of the staff, has been appointed by the War Office to be principal surgeon to a hospital shortly to be sent abroad.

Assessment of the Hospital.

For the first time in the history of the hospital, the buildings have been valued for the purpose of assessment, and the committee is glad to be able to state that the Assessment Committee has met the hospital in a very generous way.

The London Credit Drapers' Association have most generously presented the hospital with a motor ambulance worth £600.

A Mountain in Canada.

Besides the erection of a statue in London to Nurse Cavell's memory, a memorial has already been erected in the streets of Paris, and the Canadian Government has renamed a mountain in Canada Mount Cavell, in place of its former designation.

